# Prognostic and clinicopathological significance of mucin family members expression in gastric cancer: a meta-analysis

**DOI:** 10.3389/fonc.2024.1512971

**Published:** 2025-01-16

**Authors:** Shunda Wang, Yongrun Mu, Jianwei Zhang, Chengfeng Wang

**Affiliations:** Department of Pancreatic and Gastric Surgery, National Cancer Center/National Clinical Research Center for Cancer/Cancer Hospital, Chinese Academy of Medical Sciences and Peking Union Medical College, Beijing, China

**Keywords:** MUC1, MUC2, MUC5AC, MUC6, prognosis, clinicopathological characteristics

## Abstract

**Background:**

Mucin family members have been reported to be widely expressed in gastric carcinoma with diverse functions. Several important mucins exert the function of tumorigenesis or progression in gastric cancer (GC). Here, we conduct this meta-analysis to evaluate the association between mucin expression and clinicopathological features in GC.

**Methods:**

Literature searches were performed in PubMed, Embase, The Cochrane Library, and ISI Web of Science, and, finally, 28 studies met our criteria. Odds ratios or hazard ratios with 95% confidence intervals were calculated to evaluate the effect quantity. We analyzed the expression of MUC1, MUC2, MUC5AC, and MUC6 and their clinicopathological characteristics separately at the same time.

**Results:**

Twenty-eight studies that contain 4,603 patients were included in our meta-analysis. MUC1 was associated with gender, Lauren classification, depth of tumor invasion, TNM, vascular invasion, lymph metastasis, and lymphatic invasion, WHO grade, as well as the 5-year survival rate. MUC2 was significantly correlated with lymphatic invasion and WHO grade. MUC5AC was highly positive in gender, depth of tumor invasion, WHO grade, TNM, lymph metastasis, and lymphatic invasion. Moreover, cases with decreased MUC5AC expression were correlated with less 5-year survival. MUC6 was only related with lymphatic invasion.

**Conclusion:**

Our meta-analysis showed that MUC1 and MUC5AC had prognostic value in GC detected by immunohistochemistry. MUC1 and MUC5AC were also associated with some other significant clinicopathological parameters. Moreover, MUC2 and MUC6 also exert their influence in lymphatic invasion. However, further enlarged study awaits to verify our conclusion to deeply explore the role of mucin family members in GC.

## Introduction

Gastric cancer (GC) is one of common digestive system malignancies worldwide (1). Despite the prevalence of GC decreased, it remains the second most common malignant tumors in East Asia, especially in China (2). According to statistics, there are more than 300,000 newly diagnosed cases and 250,000 deaths each year (3). Gastric carcinogenesis and development are multifactorial and multistep, driven by both genetic and environmental factors, and there is a certain individual susceptibility (4). At present, there is a lack of prevention and early diagnosis methods for GC; most patients with GC are found to be advanced; and the recurrence and metastasis rate is high, which poses significant threats to human life and quality of life. At present, the main treatment of GC is surgery-based comprehensive treatment, which is more effective in combination with systemic chemotherapy (5). Despite great advances in surgical technique and chemotherapeutic agents, the mortality rate has not significantly decreased. Therefore, there is an urgent need for new therapeutic strategies to improve the prognosis of GC.

Mucins are produced by epithelial cells located on serine or threonine residues of the mucin core protein backbone (6). Mucins generally provide a safety barrier to the cell and also act as sensors of intra- and extracellular communication (7). Mucins consist of two subtypes. One is a secreted mucin (MUC1, MUC4, MUC12, and MUC16) without a transmembrane domain that could be directly secreted to the extracellular environment. The other is a membrane-bound mucin (MUC2, MUC5AC, and MUC6) with a transmembrane domain composed of numerous phosphorylation sites for signal transduction (8, 9). Mucin expression levels were shown to change at different stages of the disease, suggesting their important role in tumor initiation, progression, and metastasis. To date, it has been confirmed that mucins can participate in the progression and metastasis of different malignancies, such as pancreatic, colon, and renal cancer (7, 10, 11).

Mucin protein is main component of mucus layer of gastric. These high molecular glycoproteins protect the gastric layer form bacterial, pepsin, acid and toxic material. MUC1 is a membrane-bound mucin, whereas MUC2, MUC5AC, and MUC6 are secreted mucins. MUC5AC and MUC1 can be detected in the superficial foveolar epithelium. MUC6 mucin mainly expressed in the antral glands (12, 13). On the other hand, another secreted mucin, MUC2, is considered intestinal mucin and is rarely expressed in normal gastric mucosa but is reexpressed in the intestinal metaplasia or pre-cancer lesion (14). Mucins have shown to be closely associated with the occurrence and development of cancer. According to the pattern of gastric or intestinal mucin expression, GC is usually divided into four phenotypes: gastric, intestinal, mixed, and null (14). Other mucins’ family member can also be detected in GC but gotten less attention than the above four mucins.

Numerous studies have revealed the importance of mucin expression and its correlation with tumor malignant behavior and prognosis, but its clinicopathological significance in GC has not been fully elucidated. In some studies, MUC1 was shown to be an indicator of clinicopathological significance and used as an underlying prognostic factor of GC (15, 16). In addition, MUC2 was associated with lymphatic metastasis and the depth of tumor infiltration (17). For MUC5AC, it could be used as a poor prognostic indicator of GC (18), whereas a decrease of MUC6 might contribute to the malignant transformation of epithelial cells in GC and negatively correlated with lymphatic invasion and tumor size (19). However, there are some contradictory conclusions in other published studies regarding the relationship of mucin expression and clinicopathological parameters. Generally, meta-analysis is used to evaluate controversial clinical issues (20); therefore, we decide to elucidate this issue by systematic review and meta-analysis. Because of the limited evidence and insufficient data of single study, we develop a comprehensive meta-analysis to systematically investigate the relationship between mucin expression and clinicopathological parameters of GC, aiming to evaluate the prognostic value. We conduct the following manuscript in accordance with the preferred reporting items for systematic reviews and meta-analyses (PRISMA) reporting checklist (21).

## Methods

### Search strategy

Systematic searches of The Cochrane Library, PubMed, Embase, and ISI Web of Science, as well as China National Knowledge Infrastructure and Wan Fang databases were carried out to access original articles, which focused on mucins in the GC diagnosis, up to June 2024 without date restrictions. Keywords included (“mucin” OR “mucins” OR “MUC”) AND (“gastric cancer” OR “gastric carcinoma” OR “stomach cancer” OR “stomach carcinoma”) AND (“diagnostic” OR “diagnosis”). References cited by the selected literature were also searched for additional studies manually. PRISMA statement is performed in this meta-analysis.

### Inclusion and exclusion criteria

The inclusion criteria of our study were as follows: (1) Research studies contain the detection of MUC by immunohistochemistry in GC. (2) Cases were proven to be GC pathologically, which regarded as the golden criteria. (3) Studies included sufficient data to calculate odds ratio (OR), hazard ratio (HR), and 95% confidence interval (CI). (4) Research studies provided the relationship of clinicopathological parameters between MUC and GC. (5) Studies was published as full paper in English. Only articles wrote in English could be included.

Exclusion criteria were as follows: (1) concurrent with other malignancies or metastatic GC; (2) the gold standard was not pathological evidence; (3) the method for detecting MUC is not through immunohistochemistry; (4) unable to retract the effective data for calculation; and (5) case reports or review articles. All publications included in our study were evaluated by two independent reviewers, whereas the differences were discussed until to reach a consensus. When mistakes were found in the article, we contact the authors for further confirmation by email.

### Data extraction and quality assessment

Two reviewers independently screened eligible studies and extracted the main characters including author, country, year of publish, case numbers, MUC phenotype, detection method, and follow-up. Then, the related clinicopathological parameters between MUC and GC was listed as follows: gender, Lauren classification, tumor size, depth of tumor invasion, WHO grade, Tumor, node, metastasis (TNM), vascular invasion, lymph metastasis, lymphatic invasion, and prognosis.

Each study was assessed by two independent researchers for inclusion or exclusion, and discrepancies were settled down by a third investigator for evaluation again. Newcastle–Ottawa Scale (NOS) was used to evaluate the quality of included studies. NOS included three aspects: selection, comparability, and exposure, and scores of no less than 6 were regarded as high quality.

### Statistical analysis

Heterogeneity was assessed by Spearman correlation analysis. I^2^ > 50% indicated the presence of heterogeneity in studies, and a random-effects model (based on Mantel–Haenszel method) was adopted in the pooled analysis. Conversely, fixed–effects model (based on DerSimonian and Laird method) was used to aggregate the accuracy indicators (22, 23). The HR of 5-year survival rate was calculated from the reported data directly by number of events within 5 years after surgery was used or data reading from Kaplan–Meier survival curve. The data of survival were combined across studies using effect models for the synthesis of HR. Kaplan–Meier curves were read by Engauge Digitizer. CIs at 95% were used for all pooled data. P < 0.05 was statistically different. P-values are two-tailed. Publication bias was assessed by Deeks’ funnel plot. ReviewManager, State, and SPSS were employed to perform the analysis.

## Results

### Search result

**Figure 1:**
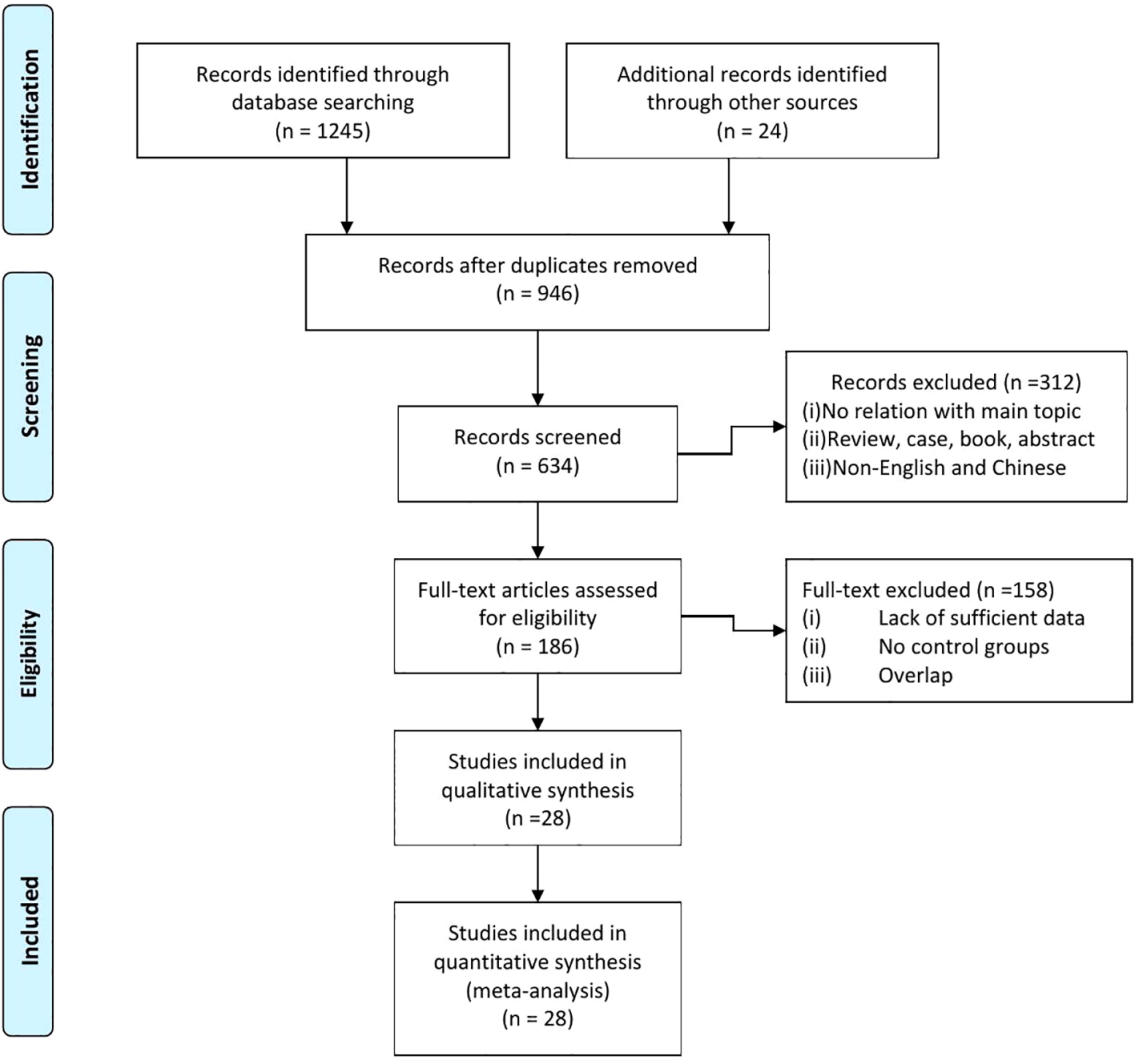
A PRISMA flow diagram depicting the selection process.

After comprehensively searching the literature of relevant studies before June 2024, 946 studies were initially screened for inclusion. As shown in **Figure 1**, we firstly search 1,269 papers. After carefully screening, we exclude the articles that dissatisfied the inclusion criteria. Articles without relationship with our main subject, abstracts, reviews, and case were excluded. The remaining 186 were further evaluated. Then, 158 were excluded because of lack of data to calculate the OR and relative risk (RR), overlapped data, and error data. Finally, there were 28 research studies included in our meta-analysis (13, 17–19, 24–47). The details of each included literature were shown in **Table 1**.

**Table 1:** The general characteristics of the included studies.

	Year	Country	Method	Age	Total number	Male	Female	Follow-up (m)	NOS
Reis	1998	UK	IHC	NA	180	105	75	NA	8
Baldus	1998	German	IHC	61.7 (34–85)	128	71	57	144	8
Lee	2001	Korea	IHC	NA	300	203	97	42 (1–60)	9
Tajima	2001	Japan	IHC	61.7 ± 13.2	136	82	54		7
Akyurek	2002	Turkey	IHC	NA	143	94	46	30(2-80)	9
Baldus	2002	German	IHC	60.8 (19.6–88.7)	200	107	93	60 (60–168)	8
Wang	2003	China	IHC	65 (32–84)	76	52	24	30 (1–58)	7
Fang	2003	China	IHC	54.6 (30–70)	46	34	12	NA	8
Tanaka	2003	Japan	IHC	NA	209	140	69	NA	7
Kocer	2004	Turkey	IHC	59.7 (22–77)	44	31	13	25 (1–79)	7
Zhang	2004	China	IHC	52.1 (25–75)	94	64	30	NA	8
Ohno	2006	Japan	IHC	63	202	142	60	NA	9
Barresi	2006	Italy	IHC	69.4 (54–77)	40	23	17	NA	9
Zheng	2006	Japan	IHC	66.3 (38–88)	225	162	63	26.1 (0.2–146.4)	8
Lee	2007	Korea	IHC	NA	98	66	32	NA	7
Li	2008	Japan	IHC	66.2 (33–85)	237	170	67	70.8 (0.2–146.4)	8
Ando	2009	Japan	IHC	63 (36–88)	102	66	36	42 (1.2–60)	9
Ilhan	2010	Turkey	IHC	NA	257	201	56	NA	8
Inagaki	2011	Japan	IHC	60 (35–84)	96	74	22	NA	8
Khattab	2011	Egypt	IHC	54.7 (37–79)	34	21	7	NA	7
Hwang	2012	Korea	IHC	56.2 (25–82)	365	248	117	NA	7
Xiao	2012	China	IHC	66.7 (38–88)	431	132	299	70.8 (0.2–146)	8
Kang	2012	Korea	IHC	NA	103	63	40	NA	7
Kim	2013	Korea	IHC	58.5	412	286	126	51.4 (1–81.5)	7
Shiratsu	2014	Japan	IHC	NA	101	78	23	NA	7
Lee	2015	Korea	IHC	61.7 ± 11.5	144	97	47	NA	7
Pyo	2015	Korea	IHC	NA	167	114	53	NA	7
Cavalcanti	2019	Italy	IHC	67.35 (34–91)	33	19	14	NA	7

NA, data not available.

### Characteristics of the included studies

Sixteen studies were included in our article for MUC1, and 15 studies focused on MUC2. Some articles are duplicated. Thirteen articles included research studies on MUC5AC. Six articles were for MUC6. Research studies were conducted in China, Turkey, UK, Korea, Italy, and other countries. There was heterogeneity in sample sizes and geographic regions of the included studies. The quality of included studies in our analysis was assessed by NOS.

### Quantitative data analysis of MUC1

**Figure 2:**
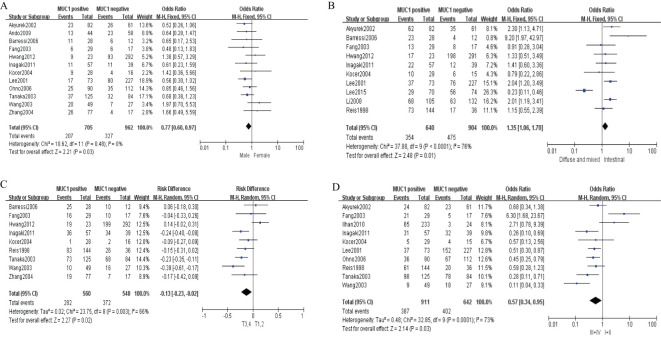
Forest plot and meta-analysis of MUC1 and clinical pathologic features, such as gender **(A)**, Lauren classification **(B)**, depth of tumor invasion **(C)**, an TNM **(D)**.

**Figure 3:**
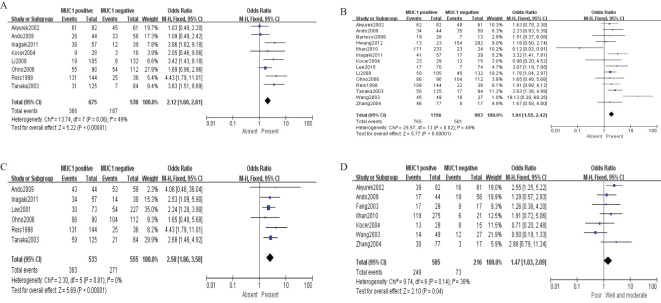
Forest plot and meta-analysis of MUC1 and clinical pathologic features, such as vascular invasion **(A)**, lymph node metastasis **(B)**, lymphatic invasion **(C)**, and WHO grade **(D)**.

**Figure 4:**
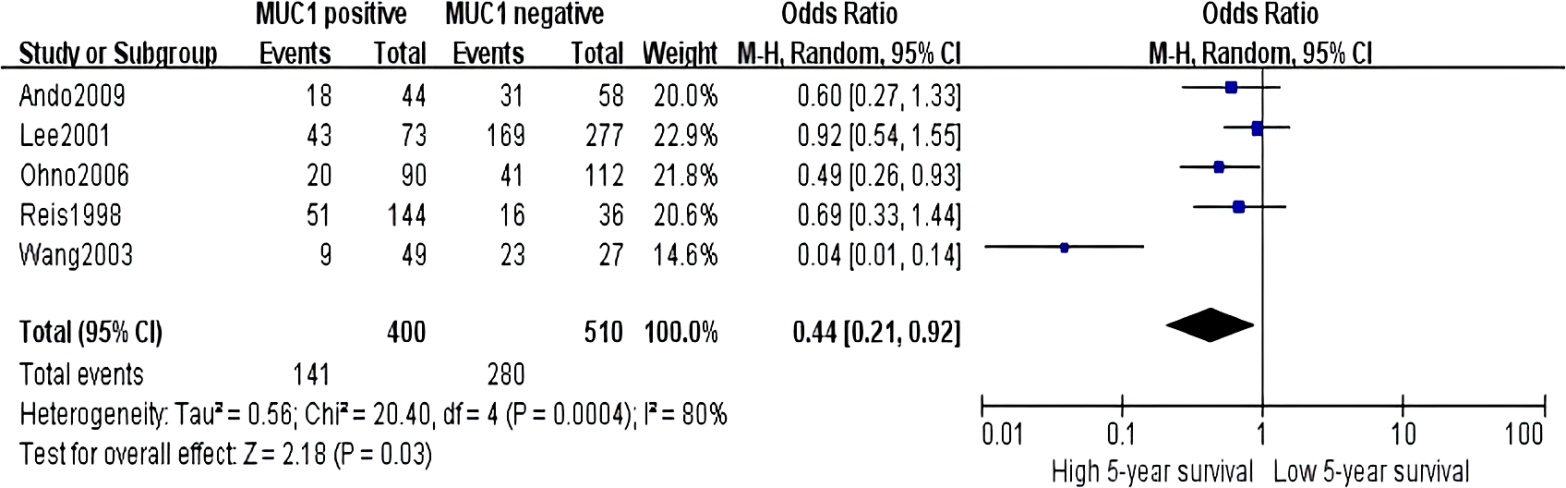
Forest plot of hazard ratio for association between decreased MUC5AC expression and 5-year survival of patients with gastric cancer.

Sixteen studies containing 2,532 patients provided data for MUC1. The putative MUC1 was associated with gender (pooled OR = 0.77, 95% CI: 0.60 to 0.97, *P* = 0.03, fixed-effects), Lauren classification (pooled OR = 1.35, 95% CI: 1.06 to 1.70, *P* = 0.01, fixed-effects), depth of tumor invasion (pooled OR = −0.13, 95% CI: −0.23 to −0.02, *P* = 0.02, fixed-effects), TNM (pooled OR = 0.57, 95% CI: 0.34 to 0.95, *P* = 0.03, fixed-effects), vascular invasion (pooled OR = 2.12, 95% CI: 1.60 to 2.81, *P* < 0.00001, fixed-effects), lymph metastasis (pooled OR = 1.94, 95% CI: 1.55 to 2.42, *P* < 0.00001, fixed-effects), lymphatic invasion (pooled OR = 2.58, 95% CI: 1.86 to 3.58, *P* < 0.00001, fixed-effects), and WHO grade (pooled OR = 1.47, 95% CI: 1.03 to 2.09, *P* = 0.04, fixed-effects) (**Figures 2**, **3**). However, MUC1 was not associated with tumor size. The T staging of GC was not dependent on tumor size but on the depth of tumor infiltration into the gastric wall. Moreover, five studies (n = 910) containing correlation between MUC1 and overall survival were included to conduct a quantitative aggregation of the survival results. Finally, the expression of MUC1 was proved to be highly associated with low 5-year survival rate in GC (pooled OR = 0.44, 95% CI: 0.21 to 0.92, *P* = 0.03, fixed-effects) (**Figure 4**).

### Quantitative data analysis of MUC2

**Figure 5:**
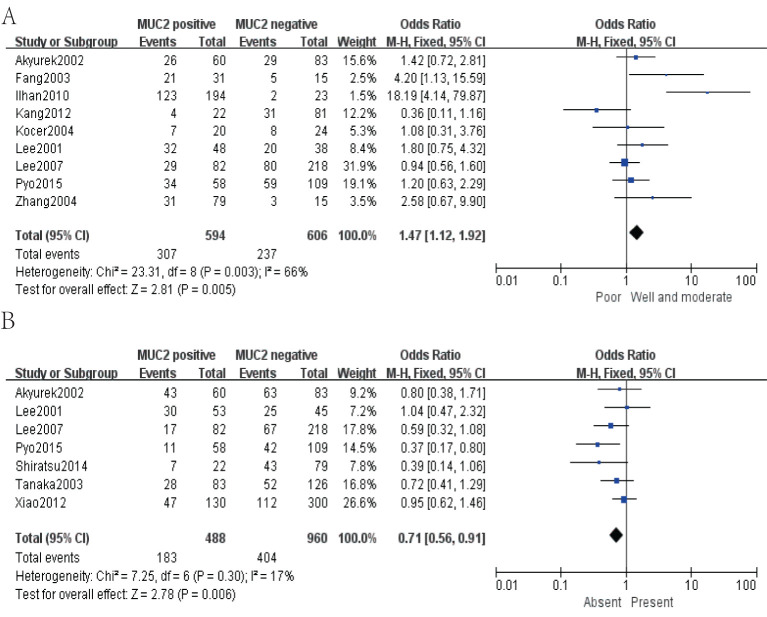
Forest plot of OR was assessed for association between MUC2 and clinical pathologic features, such as WHO grade **(A)** and lymphatic invasion **(B)**.

Fifteen studies including 2,186 cases were analyzed for the clinicopathological parameters of MUC2 expression for GC. After systematic reviews and meta-analysis, MUC2 was shown to be associated with WHO grade (pooled OR = 1.47, 95% CI: 1.12 to 1.92, *P* = 0.005, fixed-effects) and lymphatic invasion (pooled OR = 0.71, 95% CI: 0.56 to 0.91, *P* = 0.006, fixed-effects) (**Figure 5**).

### Quantitative data analysis of MUC5AC

**Figure 6:**
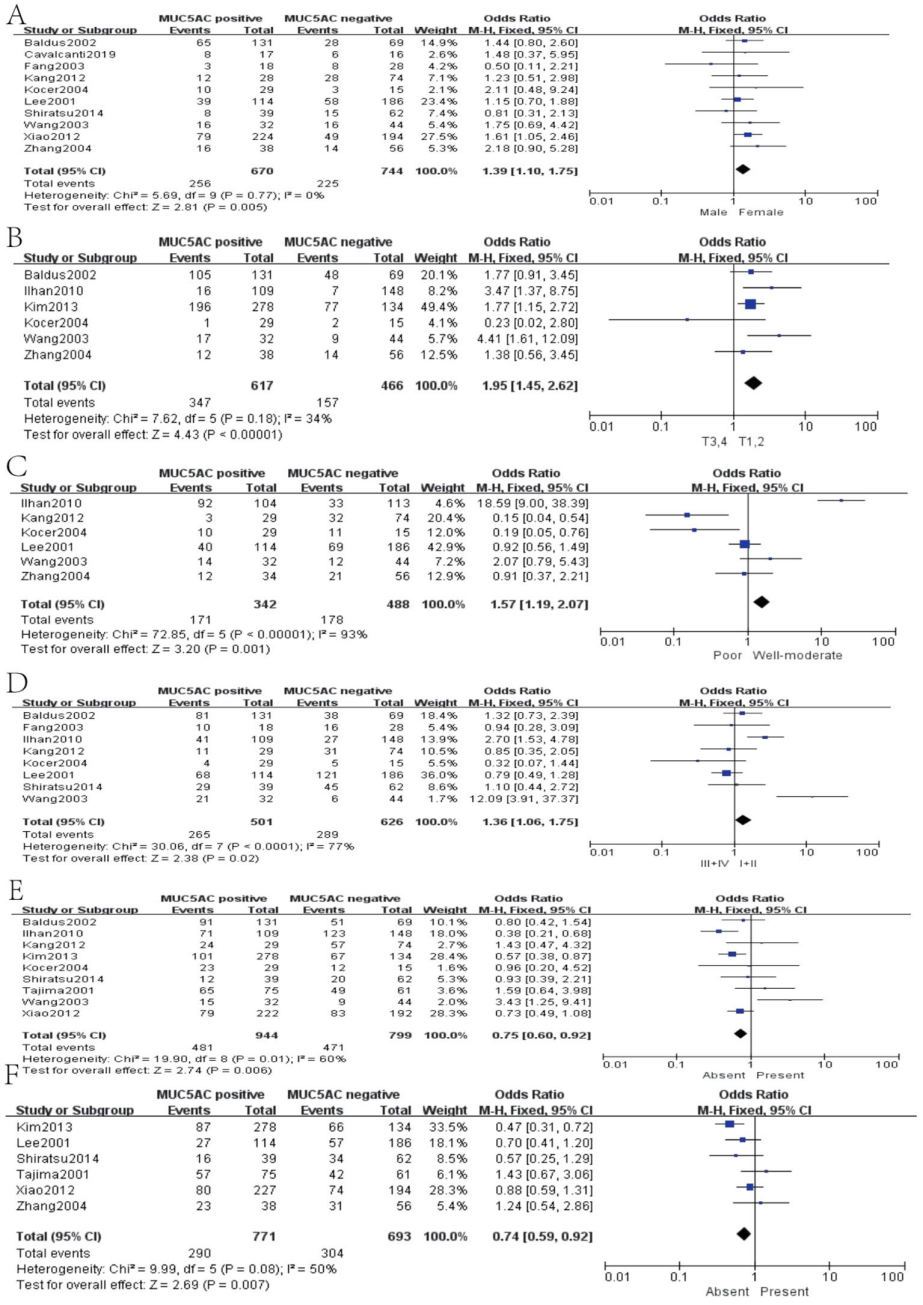
Forest plot of OR was assessed for association between MUC5 and clinical pathologic features, such as gender **(A)**, depth of tumor invasion **(B)**, WHO grade **(C)**, TNM **(D)**, lymph metastasis **(E)**, and lymphatic invasion **(F)**.

**Figure 7:**
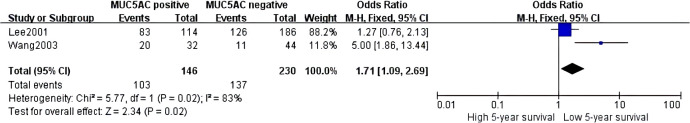
Forest plot and meta-analysis of MUC5AC and 5-year survival rate.

Thirteen studies containing 2,233 cases investigated the expression of MUC5AC and clinicopathological parameters in GC. The results of our meta-analyses manifested that MUC5AC was associated with following parameters, such as gender (pooled OR = 1.39, 95% CI: 1.10 to 1.75, *P* = 0.005, fixed-effects), depth of tumor invasion (pooled OR = 1.95, 95% CI: 1.45 to 2.62, *P* < 0.00001, fixed-effects), WHO grade (pooled OR = 1.57, 95% CI: 1.19 to 2.07, *P* = 0.001, fixed-effects), TNM (pooled OR = 1.36, 95% CI: 1.06 to 1.75, *P* = 0.02, fixed-effects), lymph metastasis (pooled OR = 0.75, 95% CI: 0.60 to 0.92, *P* = 0.006, fixed-effects), and lymphatic invasion (pooled OR = 0.74, 95% CI: 0.59 to 0.92, *P* = 0.007, fixed-effects) (**Figure 6**). Moreover, there were two articles containing 376 cases that reported the prognosis of MUC5AC, and the results proved that MUC5AC was also significantly associated with prognosis (pooled OR = 1.71, 95% CI: 1.09 to 2.69, *P* = 0.02, fixed-effects) (**Figure 7**). Perhaps due to difficulties in follow-up, survival analysis failed to be achieved in many studies.

### Quantitative data analysis of MUC6

**Figure 8:**
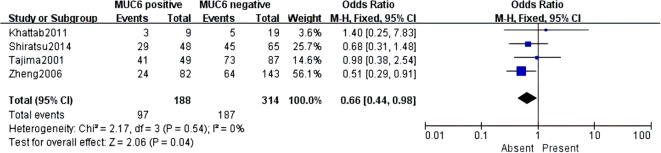
Forest plot and meta-analysis of MUC6 and lymphatic invasion.

MUC6 was mentioned in six articles, which included 836 cases. However, MUC6 was only associated with lymphatic invasion (pooled OR = 0.66, 95% CI: 0.44 to 0.98, *P* = 0.04, fixed-effects) (**Figure 8**). Although a single report on MUC6 has demonstrated its correlation with clinical indicators of GC, there was no significant difference after systematic analysis. More studies should be conducted to further explore the MUC6 expression and clinicopathological parameters of GC.

### Publication bias

**Figure 9:**
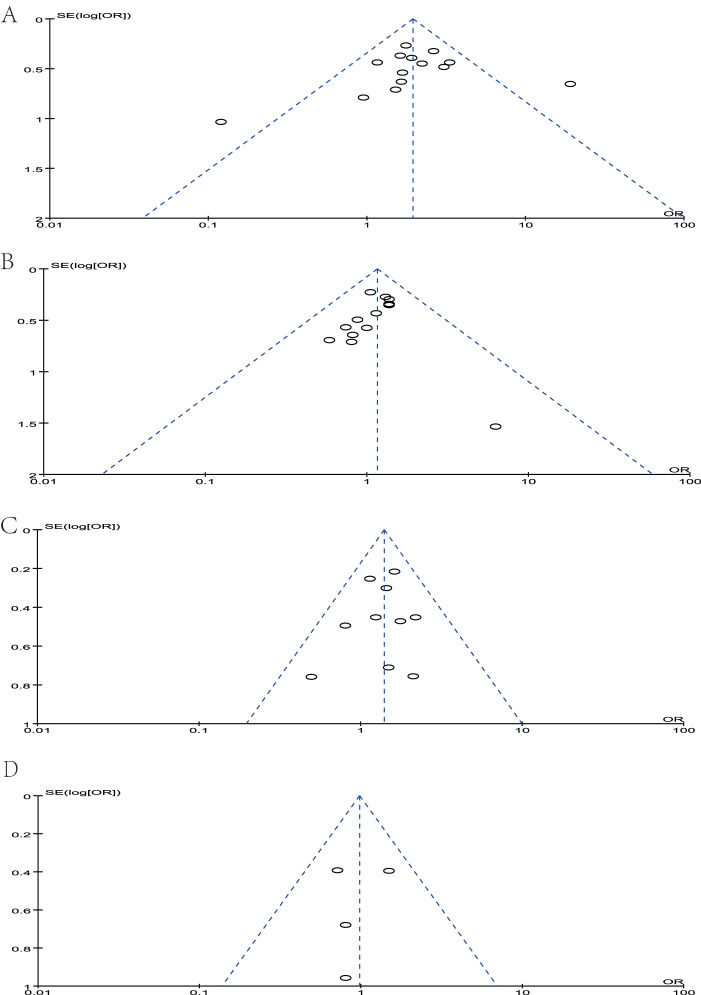
Funnel plot of studies to detect publication bias. **(A)** MUC1, **(B)** MUC2, **(C)** MUC5AC, and **(D)** MUC6.

Sensitivity analyses were performed by deleting each study included in the meta-analysis separately. No statistically significant conclusions were changed. The Newcastle–Ottawa Scale was used to evaluate the quality of included studies. Three quality parameters were used to evaluate the quality of each study: patient selection, comparability of the study groups, and assessment of the outcome. A score of 0–9 was assigned to each study, and a score of >6 indicated high study quality. We evaluate each study following the criteria of the scale, and each criterion is assigned a score of 0–3 based on the quality of the article. The NOS score of each included study was shown in **Table 1**. The publication bias was assessed by funnel plot, and no publication bias was detected in all comparisons as shown in **Figure 9**.

## Discussion

Although the incidence rate of GC has been decreasing in recent years, it is still the fifth most common malignant tumor in the world (48). Despite improvements in surgical techniques and the availability of targeted agents, the 5-year survival rate of GC is still less than 25% (49). Finding biomarkers for predicting the progression of GC is very urgent, which is beneficial for improving patient outcome. Therefore, we conducted this meta-analysis. The original intention of our study was to compare the association between several important MUC expressions and clinicopathological features of GC. To the best of our knowledge, this meta-analysis is the first study to systematically investigate MUC1, MUC2, MUC5AC, and MUC6 expression and its relationship with the patients’ clinicopathological characteristics in GC.

Mucin family is a complex and diverse group of high molecular glycoproteins, which are the main components of mucus gel on the surface of gastric mucosa. Several important mucins can be widely detected in the gastric mucosa. The main function of mucin is to resist mechanical and chemical erosion, lubricate, and resist acidity. Numerous studies have revealed the unique role of mucins in the pathogenesis of GC.

MUC1 was an extensively investigated biomarker in various cancer types, especially in gastrointestinal tract. Mostly, it exerted as a tumor promotor factor. MUC1 exerted an important role in malignant transformation, including cell proliferation, invasion, and metastatic. MUC1 was also proved to interact with different effectors such as β-catenin, receptor tyrosine kinases, and cellular-abelsongene, which were important in the pathogenesis of cancers (50). In our systematic study, MUC1 was manifested to be associated with tumor progression, and high MUC1 expression was positively correlated with vascular invasion, lymph metastasis, and lymphatic invasion. These findings indicated that positive MUC1 expression might be relevant with invasiveness of GC cells. Moreover, high MUC1 expression revealed less 5-year survival rate. The result was accordance with latest meta-analysis by Patel, proving that high MUC1 expression correlated with poorer prognosis and metastases in esophago-gastric carcinoma (51). It was reported that the polymorphism of MUC1 also influenced on the risk of GC (52). MUC1 has also been proved to reduce intracellular levels of ROS and epithelial infection and inflammation, causing the GC carcinogenesis (53). However, there are contradictory results in some studies showing that MUC1 was not an independent factor for the prognosis of patients with gastric carcinoma. This maybe because MUC1 is a prognostic factor but not an independent risk factor. It can jointly affect patient survival with other prognostic indicators. This discrepancy might also be partially attributable to their small sample size or differences of population. Profiling MUC1 expression in GC, especially combined with pre-malignant, benign, and healthy controls, can identify potential early diagnostic biomarkers. More patients and studies will be needed to confirm our findings in the future.

GC contain elements of both intestinal and diffuse types. Such heterogeneous components may distort the evaluation of the role of the mucin MUC2 in GC. There is discrepancy in expression pattern of MUC2. Such differences are not related with population source or experiment methods and maybe related with gene expression pattern. Because MUC2 was an intestinal type mucin, it was not expressed in normal gastric mucosa. However, *de novo* expressions appeared in tumors. Some study pointed out that MUC2 showed higher expression rate in diffuse type carcinomas, especially in mucinous carcinomas (30). However, other research proved that MUC2 expression was more frequent in intestinal type than the diffuse type (42). Many studies have shown that the expression pattern of mucin genes can indicate intestinal or diffuse GC subtypes, and the expression pattern changes over time. There are differences in the expression of mucin genes near tumors (41, 54). For example, MUC2 is completely absent in normal gastric mucosa and expressed in 97.8% of intestinal metaplasia subsequently in patients with GC. The positive MUC2 expression is approximately 55.4% in early GC (41). The changes of MUC2 expression during the development of GC may be mutagenic or epigenetic. The expression of MUC2 in intestinal metaplasia was higher in tumors of earlier stages. MUC2 expression in intestinal metaplasia in the neighborhood of the carcinomas may play an important role in GC (41). However, our meta-analyses also showed no significance. Maybe, it still needs further validation. Moreover, our research proved that MUC2 was also associated with lymphatic invasion, in accordance with the former research studies.

MUC5AC is a secreted mucin, which exerts a very significant function in protecting the gastric mucosa. MUC5AC also appeared to be closely linked to the development of GC. According to the former reports, MUC5AC is present in some non-tumor and precancerous diseases. In addition to serving as a protective layer and barrier, there are reports that MUC5AC could inhibit the release of tumor cells, thereby reducing invasion and metastasis (55). Moreover, *H. pylori* is important with GC. In *H. pylori*–associated gastritis, *H. pylori* disrupted the assembly of mucins and reduced gastric mucus viscosity by elevating pH (56). In intestinal metaplasia, the expression pattern of mucin peptides is altered, leading to reduced MUC5AC immunoreactivity. *H. pylori* infection was also associated with clinical outcomes of patients with GC, which could facilitate cell migration (57). Former meta-analysis proved that decreased MUC5AC was an unfavorable prognosis biomarker for patients with GC. Our research showed the same conclusion, indicating the prognostic value of MUC5AC. Moreover, in our study, MUC5AC was also proved to be related with other clinicopathological characteristics such as depth of tumor invasion, WHO grade, TNM, lymph metastasis, and lymphatic invasion. We speculated that MUC5AC might be a candidate indicator for predicting invasiveness and prognosis. Because MUC5AC is often used as a marker for surgical pathology, it is easy to detect in specimens through immunohistochemistry. Except for prognostic value, MUC5AC could also shape microbial networks in the tumor microenvironment of GC, predicting disease outcome (58).

The research on MUC6 in GC is not as extensive as other mucins. MUC6 was usually expressed aberrantly during the development of GC. The expression of MUC6 in gastric carcinomas, compared with that in adenomas and normal mucosa, was relatively in low level. This indicated that there was no change in the expression of MUC6 in gastric precancerous adenoma. As an advanced molecular event, decreased MUC6 exerts a certain function in the malignant transformation of epithelial cells during GC (12). There is no former meta-analysis to comprehensively explore MUC6 and its clinicopathological significance in GC. In our meta-analysis, MUC6 was only significantly associated with lymphatic invasion in GC, although only six articles were included. More studies of MUC6 in GC tissue are needed to be explored and conducted to deeply reveal its function. Expression of MUC6 was regulated by promotor methylation. Methylation of the MUC6 promoter may lead to significant downregulation of MUC6 in GC and promote the progression of GC (59). Further investigation on MUC6 should be focused on epigenetic regulation.

Taken together, there were several unsolvable limitations in this meta-analysis that need to be pointed out. Firstly, the included studies were non-retrospective, which might influence the credibility of meta-analysis. However, it was hard to conduct randomized controlled trial (RCT) research about MUC pathological stain and clinicopathological parameters in GC. The strongest evidence for this problem should still be from prospective cohort studies. Moreover, heterogeneity between studies was low for most of the dichotomous variables examined but was marked for all the continuous variables. There were significant variabilities such as definitions, inclusion or exclusion criteria, Immunohistochemistry (IHC) staining, and IHC interpretation. It was impossible to match all patient cohorts for gender, age, preoperative therapy, and previous history. All these factors might contribute to the high heterogeneity between studies. Moreover, selection bias among the included literature was inevitable. Although the interpretation of MUC expression was dependent on the gold standard, there was a lack of blinding. On the other hand, most studies used similar IHC techniques, but the different antibodies used in the staining caused in the variation of account in the results yielded. Finally, small sample size might contribute to the heterogeneity among studies. The enlarged number of patients might eliminate the between-study heterogeneity.

## Conclusion

This meta-analysis focused on investigating the clinicopathological significance of various mucin expressions in GC. This was definitely the first meta-analysis to evaluate several mucins and GC’s clinicopathological factors. MUC1, MUC2, MUC5AC, and MUC6 were the most commonly analyzed mucins across cancer types according to this literature review. We identified that high MUC1 expression was significantly associated with GC invasiveness and worse overall survival. MUC2, MUC5AC, and MUC6 may also contribute to carcinogenesis and malignant progression. There is an urgent need to further investigate the function and mechanism of different mucins in GC. Further clinical studies are needed to confirm the role of MUC family members in GC.

## Data Availability

The original contributions presented in the study are included in the article/supplementary material. Further inquiries can be directed to the corresponding author.

## References

[1] Van Cutsem E Sagaert X Topal B Haustermans K Prenen H . Gastric cancer. Lancet. (2016) 388:2654–64. doi: 10.1016/S0140-6736(16)30354-3 27156933

[2] Ferlay J Shin HR Bray F Forman D Mathers C Parkin DM . Estimates of worldwide burden of cancer in 2008: globocan 2008. Int J Cancer. (2010) 127:2893–917. doi: 10.1002/ijc.25516 21351269

[3] Siegel RL Giaquinto AN Jemal A . Cancer statistics, 2024. CA: Cancer J For Clin. (2024) 74:12–49. doi: 10.3322/caac.21820 38230766

[4] McLean MH El-Omar EM . Genetics of gastric cancer. Nat Rev Gastroenterol Hepatol. (2014) 11:664–74. doi: 10.1038/nrgastro.2014.143 25134511

[5] Sonkin D Thomas A Teicher BA . Cancer treatments: past, present, and future. Cancer Genet. (2024) 286-287:18–24. doi: 10.1016/j.cancergen.2024.06.002 38909530 PMC11338712

[6] Chakraborty S Bonthu N Swanson BJ Batra SK . Role of mucins in the skin during benign and Malignant conditions. Cancer Lett. (2011) 301:127–41. doi: 10.1016/j.canlet.2010.11.004 PMC3232046 21146919

[7] Byrd JC Bresalier RS . Mucins and mucin binding proteins in colorectal cancer. Cancer Metastasis Rev. (2004) 23:77–99. doi: 10.1023/a:1025815113599 15000151

[8] Huang J Che MI Huang YT Shyu MK Huang YM Wu YM . Overexpression of muc15 activates extracellular signal-regulated kinase 1/2 and promotes the oncogenic potential of human colon cancer cells. Carcinogenesis. (2009) 30:1452–8. doi: 10.1093/carcin/bgp137 19520792

[9] Singh PK Hollingsworth MA . Cell surface-associated mucins in signal transduction. Trends Cell Biol. (2006) 16:467–76. doi: 10.1016/j.tcb.2006.07.006 16904320

[10] Wang S Li J You L Dai M Zhao Y . High expression of muc15 is correlated with poor prognosis of pancreatic cancer and promotes migration, invasion, and chemo-resistance *in vitro* . Med Sci Monit. (2020) 26:e926432. doi: 10.12659/MSM.926432 33051432 PMC7570823

[11] Yue Y Hui K Wu S Zhang M Que T Gu Y . Muc15 inhibits cancer metastasis via pi3k/akt signaling in renal cell carcinoma. Cell Death Dis. (2020) 11:336. doi: 10.1038/s41419-020-2518-9 32382053 PMC7205982

[12] Ho SB Shekels LL Toribara NW Kim YS Lyftogt C Cherwitz DL . Mucin gene expression in normal, preneoplastic, and neoplastic human gastric epithelium. Cancer Res. (1995) 55:2681–90. 7780985

[13] Reis CA David L Seixas M Burchell J Sobrinho-Simoes M . Expression of fully and under-glycosylated forms of muc1 mucin in gastric carcinoma. Int J Cancer. (1998) 79:402–10. doi: 10.1002/(sici)1097-0215(19980821)79:4<402::aid-ijc16>3.0.co;2-6 9699534

[14] Wakatsuki K Yamada Y Narikiyo M Ueno M Takayama T Tamaki H . Clinicopathological and prognostic significance of mucin phenotype in gastric cancer. J Surg Oncol. (2008) 98:124–9. doi: 10.1002/jso.21093 18521835

[15] Saeki N Sakamoto H Yoshida T . Mucin 1 gene (Muc1) and gastric-cancer susceptibility. Int J Mol Sci. (2014) 15:7958–73. doi: 10.3390/ijms15057958 PMC4057712 24810688

[16] Yonezawa S Kitajima S Higashi M Osako M Horinouchi M Yokoyama S . A novel anti-muc1 antibody against the muc1 cytoplasmic tail domain: use in sensitive identification of poorly differentiated cells in adenocarcinoma of the stomach. Gastric Cancer. (2012) 15:370–81. doi: 10.1007/s10120-011-0125-2 PMC3477479 22237656

[17] Pyo JS Sohn JH Kang G Kim DH Kim K Do IG . Muc2 expression is correlated with tumor differentiation and inhibits tumor invasion in gastric carcinomas: A systematic review and meta-analysis. J Pathol Transl Med. (2015) 49:249–56. doi: 10.4132/jptm.2015.03.27 PMC4440937 26018517

[18] Baldus SE Monig SP Arkenau V Hanisch FG Schneider PM Thiele J . Correlation of muc5ac immunoreactivity with histopathological subtypes and prognosis of gastric carcinoma. Ann Surg Oncol. (2002) 9:887–93. doi: 10.1007/BF02557526 12417511

[19] Zheng H Takahashi H Nakajima T Murai Y Cui Z Nomoto K . Muc6 down-regulation correlates with gastric carcinoma progression and a poor prognosis: an immunohistochemical study with tissue microarrays. J Cancer Res Clin Oncol. (2006) 132:817–23. doi: 10.1007/s00432-006-0135-3 PMC12161041 16807756

[20] Li R Mukherjee MB Jin Z Liu H Lin K Liu Q . The potential effect of general anesthetics in cancer surgery: meta-analysis of postoperative metastasis and inflammatory cytokines. Cancers. (2023) 15. doi: 10.3390/cancers15102759 PMC10216624 37345096

[21] Moher D Liberati A Tetzlaff J Altman DG Group P . Preferred reporting items for systematic reviews and meta-analyses: the prisma statement. PloS Med. (2009) 6:e1000097. doi: 10.1371/journal.pmed.1000097 19621072 PMC2707599

[22] Higgins JP Thompson SG Deeks JJ Altman DG . Measuring inconsistency in meta-analyses. BMJ. (2003) 327:557–60. doi: 10.1136/bmj.327.7414.557 PMC192859 12958120

[23] Lau J Ioannidis JP Schmid CH . Quantitative synthesis in systematic reviews. Ann Intern Med. (1997) 127:820–6. doi: 10.7326/0003-4819-127-9-199711010-00008 9382404

[24] Inagaki Y Tang W Xu H Nakata M Mafune K-I Konishi T . Sustained aberrant localization of kl-6 mucin and beta-catenin at the invasion front of human gastric cancer cells. Anticancer Res. (2011) 31:535–42. 21378335

[25] Tanaka M Kitajima Y Sato S Miyazaki K . Combined evaluation of mucin antigen and E-cadherin expression may help select patients with gastric cancer suitable for minimally invasive therapy. Br J Surg. (2003) 90(1):95–101. doi: 10.1002/bjs.4014 12520583

[26] Hwang I Kang YN Kim JY Do YR Song HS Park KU . Prognostic significance of membrane-associated mucins 1 and 4 in gastric adenocarcinoma. Exp Ther Med. (2012) 4:311–6. doi: 10.3892/etm.2012.598 PMC3460289 23139719

[27] Ando H Aihara R Ohno T Ogata K Mochiki E Kuwano H . Prognostic significance of the expression of muc1 and collagen type iv in advanced gastric carcinoma. Br J Surg. (2009) 96:901–9. doi: 10.1002/bjs.6635 19591170

[28] Lee SH Park HK Kim JH Han HS . Significance of muc1 expression in biopsy specimens of submucosal invasive gastric carcinoma: the association with lymph node metastasis. Oncol Lett. (2015) 10:1437–43. doi: 10.3892/ol.2015.3483 PMC4533718 26622686

[29] Li X-H Zheng H-C Wang Z-G Takahashi H Yang X-H Guan Y-F . The clinicopathological and prognostic significance of muc-1 expression in Japanese gastric carcinomas: an immunohistochemical study of tissue microarrays. Anticancer Res. (2008) 28:1061–7. 18507055

[30] Barresi V Vitarelli E Grosso M Tuccari G Barresi G . Relationship between immunoexpression of mucin peptide cores muc1 and muc2 and lauren’s histologic subtypes of gastric carcinomas. Eur J Histochem. (2006) 50:301–9. 17213039

[31] Zhang H-K Zhang Q-M Zhao T-H Li Y-Y Yi Y-F . Expression of mucins and E-cadherin in gastric carcinoma and their clinical significance. World J Gastroenterol. (2004) 10:3044–7. doi: 10.3748/wjg.v10.i20.3044 PMC4576269 15378790

[32] Wang RQ Fang DC . Alterations of muc1 and muc3 expression in gastric carcinoma: relevance to patient clinicopathological features. J Clin Pathol. (2003) 56:378–84. doi: 10.1136/jcp.56.5.378 PMC1769961 12719460

[33] İlhan Ö Han Ü Önal B Çelık SY . Prognostic significance of muc1, muc2 and muc5ac expressions in gastric carcinoma. Turk J Gastroenterol. (2010) 21:345–52. doi: 10.4318/tjg.2010.0119 21331986

[34] Ohno T Aihara R Kamiyama Y Mochiki E Asao T Kuwano H . Prognostic significance of combined expression of muc1 and adhesion molecules in advanced gastric cancer. Eur J Cancer. (2006) 42:256–63. doi: 10.1016/j.ejca.2005.10.017 16356709

[35] Kocer B Soran A Kiyak G Erdogan S Eroglu A Bozkurt B . Prognostic significance of mucin expression in gastric carcinoma. Dig Dis Sci. (2004) 49:954–64. doi: 10.1023/B:DDAS.0000034554.96191.66 15309883

[36] Wang J-Y Chang C-T Hsieh J-S Lee L-W Huang T-J Chai C-Y . Role of muc1 and muc5ac expressions as prognostic indicators in gastric carcinomas. J Surg Oncol. (2003) 83:253–60. doi: 10.1002/jso.10222 12884239

[37] Lee HS Lee HK Kim HS Yang HK Kim YI Kim WH . Muc1, muc2, muc5ac, and muc6 expressions in gastric carcinomas: their roles as prognostic indicators. Cancer. (2001) 92:1427–34. doi: 10.1002/1097-0142(20010915)92:6<1427::AID-CNCR1466>3.0.CO;2-L 11745219

[38] Akyürek N Akyol G Dursun A Yamaç D Günel N . Expression of muc1 and muc2 mucins in gastric carcinomas: their relationship with clinicopathologic parameters and prognosis. Pathol Res Pract. (2002) 198:665–74. doi: 10.1078/0344-0338-00318 12498221

[39] Kang H An H-J Song J-Y Kim T-H Heo J-H Ahn D-H . Notch3 and jagged2 contribute to gastric cancer development and to glandular differentiation associated with muc2 and muc5ac expression. Histopathology. (2012) 61:576–86. doi: 10.1111/j.1365-2559.2012.04274.x 22691042

[40] Xiao L-J Zhao S Zhao E-H Zheng X Gou W-F Xing Y-N . Clinicopathological and prognostic significance of muc-2, muc-4 and muc-5ac expression in Japanese gastric carcinomas. Asian Pac J Cancer Prev. (2012) 13:6447–53. doi: 10.7314/APJCP.2012.13.12.6447 23464473

[41] Lee H-W Yang DH Kim HK Lee BH Choi KC Choi YH . Expression of muc2 in gastric carcinomas and background mucosae. J Gastroenterol Hepatol. (2007) 22:1336–43. doi: 10.1111/j.1440-1746.2007.04939.x 17559374

[42] Baldus SE Zirbes TK Engel S Hanisch FG Mönig SP Lorenzen J . Correlation of the immunohistochemical reactivity of mucin peptide cores muc1 and muc2 with the histopathological subtype and prognosis of gastric carcinomas. Int J Cancer. (1998) 79:133–8. doi: 10.1002/(SICI)1097-0215(19980417)79:2<133::AID-IJC6>3.0.CO;2-U 9583726

[43] Khattab A-ZM Nasif WA Lotfy M . Muc2 and muc6 apomucins expression in human gastric neoplasm: an immunohistochemical analysis. Med Oncol. (2011) 28 Suppl 1:S207–S13. doi: 10.1007/s12032-010-9699-5 20878553

[44] Shiratsu K Higuchi K Nakayama J . Loss of gastric gland mucin-specific O-glycan is associated with progression of differentiated-type adenocarcinoma of the stomach. Cancer Sci. (2014) 105:126–33. doi: 10.1111/cas.12305 PMC4317868 24138592

[45] Tajima Y Shimoda T Nakanishi Y Yokoyama N Tanaka T Shimizu K . Gastric and intestinal phenotypic marker expression in gastric carcinomas and its prognostic significance: immunohistochemical analysis of 136 lesions. Oncology. (2001) 61:212–20. doi: 10.1159/000055377 11574777

[46] Kim SM Kwon CH Shin N Park DY Moon HJ Kim GH . Decreased muc5ac expression is associated with poor prognosis in gastric cancer. Int J Cancer. (2014) 134:114–24. doi: 10.1002/ijc.28345 23801416

[47] Cavalcanti E De Michele F Lantone G Panarese A Caruso ML . Mucin phenotype of differentiated early gastric cancer: an immunohistochemistry study supporting therapeutic decision making. Cancer Manag Res. (2019) 11:5047–54. doi: 10.2147/CMAR.S193994 PMC6589520 31354341

[48] de Martel C Forman D Plummer M . Gastric cancer: epidemiology and risk factors. Gastroenterol Clin North Am. (2013) 42:219–40. doi: 10.1016/j.gtc.2013.01.003 23639638

[49] Amedei A Benagiano M della Bella C Niccolai E D’Elios MM . Novel immunotherapeutic strategies of gastric cancer treatment. J BioMed Biotechnol. (2011) 2011:437348. doi: 10.1155/2011/437348 22253528 PMC3255571

[50] Tong X Dong C Liang S . Mucin1 as a potential molecule for cancer immunotherapy and targeted therapy. J Cancer. (2024) 15:54–67. doi: 10.7150/jca.88261 38164273 PMC10751670

[51] Patel NM Geropoulos G Patel PH Bhogal RH Harrington KJ Singanayagam A . The role of mucin expression in the diagnosis of oesophago-gastric cancer: A systematic literature review. Cancers (Basel). (2023) 15. doi: 10.3390/cancers15215252 PMC10650431 37958425

[52] Liu P Zeng M . Role of muc1 rs4072037 polymorphism in gastric cancer: A meta-analysis. Int J Clin Exp Pathol. (2020) 13:465–72. PMC7137027 32269683

[53] Yin L Li Y Ren J Kuwahara H Kufe D . Human muc1 carcinoma antigen regulates intracellular oxidant levels and the apoptotic response to oxidative stress. J Biol Chem. (2003) 278:35458–64. doi: 10.1074/jbc.M301987200 12826677

[54] Kim DH Shin N Kim GH Song GA Jeon TY Kim DH . Mucin expression in gastric cancer: reappraisal of its clinicopathologic and prognostic significance. Arch Pathol Lab Med. (2013) 137:1047–53. doi: 10.5858/arpa.2012-0193-OA 23899060

[55] Corfield AP Myerscough N Longman R Sylvester P Arul S Pignatelli M . Mucins and mucosal protection in the gastrointestinal tract: new prospects for mucins in the pathology of gastrointestinal disease. Gut. (2000) 47:589–94. doi: 10.1136/gut.47.4.589 PMC1728059 10986224

[56] Celli JP Turner BS Afdhal NH Keates S Ghiran I Kelly CP . Helicobacter pylori moves through mucus by reducing mucin viscoelasticity. Proc Natl Acad Sci United States America. (2009) 106:14321–6. doi: 10.1073/pnas.0903438106 PMC2732822 19706518

[57] Ou L Liu H Peng C Zou Y Jia J Li H . Helicobacter pylori infection facilitates cell migration and potentially impact clinical outcomes in gastric cancer. Heliyon. (2024) 10:e37046. doi: 10.1016/j.heliyon.2024.e37046 39286209 PMC11402937

[58] Oosterlinck B Ceuleers H Arras W De Man JG Geboes K De Schepper H . Mucin-microbiome signatures shape the tumor microenvironment in gastric cancer. Microbiome. (2023) 11:86. doi: 10.1186/s40168-023-01534-w 37085819 PMC10120190

[59] Shi D Xi X-X . Regulation of muc6 methylation correlates with progression of gastric cancer. Yonsei Med J. (2021) 62:1005–15. doi: 10.3349/ymj.2021.62.11.1005 PMC8542475 34672134

